# Microstructures of HfO_x_ Films Prepared via Atomic Layer Deposition Using La(NO_3_)_3_·6H_2_O Oxidants

**DOI:** 10.3390/ma14237478

**Published:** 2021-12-06

**Authors:** Seon Yong Kim, Yong Chan Jung, Sejong Seong, Taehoon Lee, In-Sung Park, Jinho Ahn

**Affiliations:** 1 Division of Materials Science and Engineering, Hanyang University, Seoul 04763, Korea; tjsdyd93@hanyang.ac.kr (S.Y.K.); Yongchan.Jung@utdallas.edu (Y.C.J.); sejongseong@gmail.com (S.S.); lth911218@gmail.com (T.L.); 2 Department of Materials Science and Engineering, The University of Texas at Dallas, Richardson, TX 75080, USA; 3 Institute of Nano Science and Technology, Hanyang University, Seoul 04763, Korea

**Keywords:** hafnium oxide film, atomic layer deposition, MOSFET

## Abstract

Hafnium oxide (HfO_x_) films have a wide range of applications in solid-state devices, including metal–oxide–semiconductor field-effect transistors (MOSFETs). The growth of HfO_x_ films from the metal precursor tetrakis(ethylmethylamino) hafnium with La(NO_3_)_3_·6H_2_O solution (LNS) as an oxidant was investigated. The atomic layer deposition (ALD) conditions were optimized, and the chemical state, surface morphology, and microstructure of the prepared films were characterized. Furthermore, to better understand the effects of LNS on the deposition process, HfO_x_ films deposited using a conventional oxidant (H_2_O) were also prepared. The ALD process using LNS was observed to be self-limiting, with an ALD temperature window of 200–350 °C and a growth rate of 1.6 Å per cycle, two times faster than that with H_2_O. HfO_x_ films deposited using the LNS oxidant had smaller crystallites than those deposited using H_2_O, as well as more suboxides or defects because of the higher number of grain boundaries. In addition, there was a difference in the preferred orientations of the HfO_x_ films deposited using LNS and H_2_O, and consequently, a difference in surface energy. Finally, a film growth model based on the surface energy difference was proposed to explain the observed growth rate and crystallite size trends.

## 1. Introduction

Recently, hafnium oxide (HfO_x_) thin films have been studied as promising electronic materials for a wide range of solid-state device applications. The excellent insulating and dielectric properties of HfO_x_ enable its application in semiconductor devices. Thin films based on HfO_x_ have substituted SiO_2_ as the material of choice for the gate dielectric layer in metal–oxide–semiconductor field-effect transistors (MOSFETs) because of their high dielectric constant, wide band gap, large band offset, and good thermodynamic stability on Si wafers [1]. More recently, HfO_x_ has been widely studied as a candidate insulating layer in resistors with metal–insulator–metal structures, which are used in non-volatile resistive switching memory [2]. Furthermore, HfO_x_ doped with La and Zr has attracted attention for use in CMOS-compatible ferroelectric devices [3,4]. The dopants distort the structure of HfO_x_, generating a ferroelectric polar orthorhombic structure.

With the continued reduction in size and increase in complexity of semiconductor devices, a need for the fabrication of ultrathin films with precisely controlled thickness on three-dimensional device structures is becoming apparent. To meet this requirement, atomic layer deposition (ALD) is one possible thin film fabrication method [5]. To fabricate metal oxide films, a typical ALD cycle consists of four steps: pulsing the metal precursor, purging the remnant with inert gas, pulsing the oxidant, and purging the remnant with inert gas. ALD via the above basic process has the advantage of offering precisely controlled of ultrathin layers with good uniformity, as well as excellent conformal coating of surfaces with intricate structures [6].

However, because of the extremely slow growth rate in ALD, low productivity is a serious disadvantage. To enhance the throughput of the ALD method, many studies have been focused on developing batch-type ALD and spatial ALD [7,8]. In particular, both metal precursors and oxidants can modulate the characteristics of metal oxide films; that is, the choice of these materials influences the growth rate, ALD temperature window, crystalline structure, contamination, and dielectric and electrical properties. Various oxidants have been used to prepare ALD oxide films, such as H_2_O, H_2_O_2_, O_3_, and plasma-based radical oxygen [1,9]. In addition to the precursor and oxidant, the catalyst can strongly affect the deposition properties and material characteristics of films grown by ALD. To fabricate ZrO_2_ films, Oh et al. used La(NO_3_)_3_∙6H_2_O solution instead of H_2_O as an oxidant and compared the crystalline phase, grain size, and surface roughness of the resulting ZrO_2_ films [10]. Interestingly, use of the La(NO_3_)_3_∙6H_2_O solution increased the ZrO_2_ film growth rate because of a catalytic effect of the La-based oxidant. In addition, HfO_2_ films deposited with La(NO_3_)_3_∙6H_2_O solution instead of H_2_O exhibited modified resistive switching characteristics [11]. However, in those studies, characterization of the specific ALD processes involved when a solution oxidant is used, in terms of self-saturation, ALD temperature window, and growth linearity, was lacking. In addition, the suggested mechanism did not adequately explain the origins of the microstructural differences observed. Accordingly, in this study, a La(NO_3_)_3_∙6H_2_O solution was used as an oxidant for ALD, with the aim of optimizing the ALD process.

In this work, we focused on the use of La(NO_3_)_3_∙6H_2_O as a catalytic oxidant in the ALD of HfO_x_ films. The properties of these films were compared with those of HfO_x_ films fabricated via ALD using H_2_O as an oxidant; the film thickness was monitored as a function of precursor and oxidant pulse time, deposition temperature, and the number of ALD cycles. The chemical, surface morphological, and structural properties of the deposited HfO_x_ films were analyzed by X-ray photoelectron spectroscopy (XPS), atomic force microscopy (AFM), grazing-incidence X-ray diffraction (GI-XRD), and transmission electron microscopy (TEM). Furthermore, a deposition mechanism was proposed to explain the difference between the growth properties and microstructures of HfO_2_ films fabricated using the La(NO_3_)_3_∙6H_2_O solution and H_2_O.

## 2. Materials and Methods

### 2.1. HfO_x_ Film Fabrication

HfO_x_ thin films were deposited on Si(100) substrates by ALD using tetrakis (ethylmethylamino) hafnium (TEMAH) as the metal precursor and 40 wt% La(NO_3_)_3_·6H_2_O solution (LNS) as an oxidant. To better understand the effects of LNS, HfO_x_ thin films were also prepared using H_2_O as the oxidant, and the deposition and material properties of these were compared with those of the thin films prepared using LNS. Prior to the deposition of the HfO_x_ thin films, p-type Si(100) substrates were cleaned in a dilute HF solution to remove the native oxide film. The metal precursor, TEMAH, was volatilized at 60 °C and delivered into a vacuum chamber filled with a pure N_2_ carrier gas (>99.999%). The liquid oxidant (LNS or H_2_O) was vaporized at room temperature, and the vaporized oxidant was introduced into the chamber without any carrier gas. A cycle of ALD consisted of the following four steps: (1) pulsing TEMAH with N_2_ carrier gas, (2) purging for 60 s with N_2_ gas (>99.999%), (3) pulsing the oxidant (LNS or H_2_O), and (4) purging for 60 s with N_2_ gas (>99.999%). To optimize the HfO_x_ ALD process involving LNS, the substrate temperature (200–400 °C), TEMAH pulse time, and LNS pulse time were varied.

### 2.2. Analyses of HfO_x_ Thin Films Properties

The thickness of the film deposited on the Si substrate was measured using spectroscopic ellipsometry (UVISEL, Horiba, Kyoto, Japan). XPS (Theta Probe, Thermo Fisher Scientific Co., Waltham, MA, USA) was used to analyze the chemical bonding of the HfO_x_ films and to ascertain the presence or absence of La in the HfO_x_ films. The morphological properties of the deposited films were characterized by AFM (XE-100, Park Systems, Suwon, Korea). Furthermore, the crystalline phase and crystallite size and orientation were determined by GI-XRD (SmartLab, Rigaku, Tokyo, Japan) and TEM (Tecnai F20 G^2^, FEI, Hillsboro, OR, USA). For the cross-sectional TEM image, a sample was prepared using the focused ion beam system, and for the plan view TEM image, samples were prepared using the ion milling system.

## 3. Results and Discussion

### 3.1. ALD Process for HfO_x_ Film Growth Using LNS

**Figure 1:**
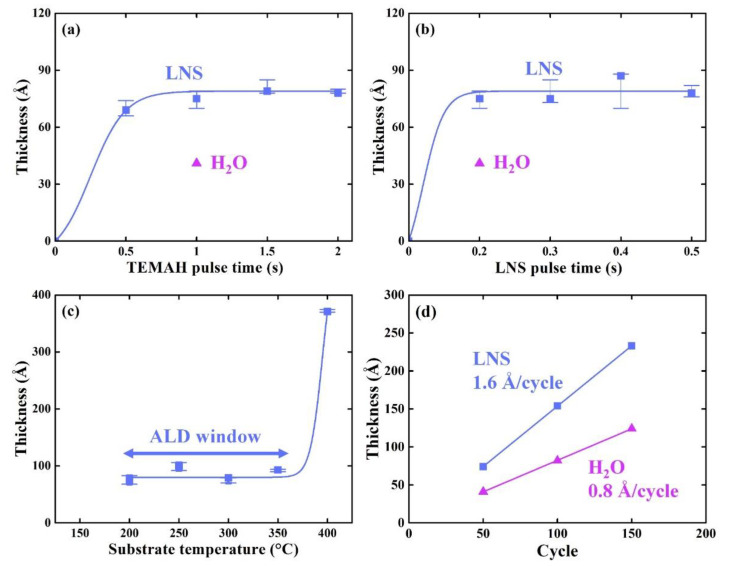
Thickness of HfO_x_ films grown using LNS as a function of (**a**) TEMAH pulse time, (**b**) LNS pulse time, (**c**) substrate temperature, and (**d**) ALD cycle. Data for films grown using H_2_O is shown in purple for comparison in (**a**,**b**,**d**). The growth rates were 1.6 Å per cycle for the process involving LNS and 0.8 Å per cycle for that involving H_2_O.

The characteristics of HfO_x_ thin films deposited using LNS as an oxidant were investigated by varying several process parameters (Figure 1a–d). Figure 1a, b show film growth saturation curves as a function of Hf-precursor (TEMAH) and LNS pulse times, respectively, with a substrate temperature of 300 ℃ over 50 cycles. For LNS pulse times of ≥0.2 s, the self-limiting characteristic of the reaction was apparent, as shown in Figure 1a; for these experiments, TEMAH was injected into the process chamber for more than 1 s. Figure 1b shows the film growth saturation curve as a function of LNS pulse times greater than 0.2 s. After examining Figure 1a and b, the optimized HfO_x_ film deposition process conditions were determined to be a substrate temperature of 300 °C, Hf-precursor pulse time of 1 s, and LNS pulse time of 0.2 s. Note that when H_2_O was used as an oxidant instead of LNS, the oxidant pulse time was the same (0.2 s).

The temperature window for ALD with the TEMAH precursor and LNS oxidant was characterized by measuring the thickness of deposited HfO_x_ films after 50 ALD cycles as a function of the substrate temperature from 200 °C to 400 °C (Figure 1c). The ALD process temperature window is defined as the temperature range over which a constant thickness is deposited, which was determined to be below 350 °C in this study. The temperature window for the process involving LNS was found to differ from that of the process with H_2_O, which was in the range of 200 °C to 400 °C [12].

HfO_x_ films were deposited under the optimized ALD conditions—a TEMAH pulse time of 1 s, LNS pulse time of 0.2 s, and temperature of 300 °C—using various numbers of ALD cycles. As can be seen in Figure 1d, the thickness of the HfO_x_ thin films increased linearly with the number of cycles. A HfO_x_ growth rate of 1.6 Å per cycle was obtained with the use of the LNS oxidant. Interestingly, this rate was two times faster than that measured when H_2_O was used with the same pulse time (0.8 Å per cycle).

### 3.2. Microstructure of HfO_x_ Films Prepared Using LNS

**Figure 2:**
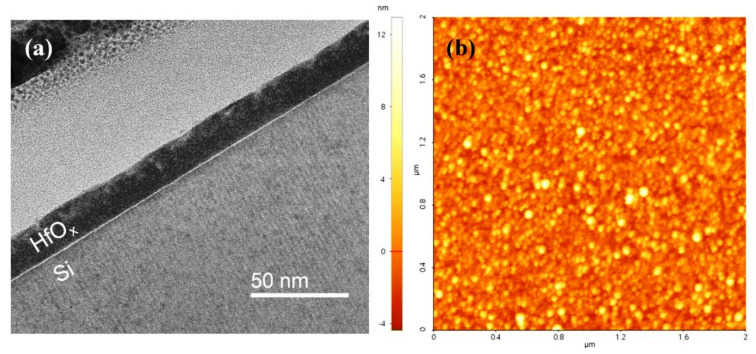
(**a**) TEM image and (**b**) AFM image of 20-nm-thick HfO_x_ film deposited on Si substrate (RMS roughness, 1.74 nm).

Figure 2a shows a cross-sectional TEM image of HfO_x_ deposited on an Si substrate after 125 cycles of the optimized ALD process using LNS (TEMAH pulse time, 1 s; LNS pulse time, 0.2 s; 300 °C). The TEM image, as expected, clearly depicts an interface region consisting of native oxide (SiO_x_). The thickness of the HfO_x_ film was uniform, and the average thickness was 20 nm. The surface morphology of the HfO_x_ film is apparent in the 2 × 2 μm^2^ AFM image in Figure 2b. The root mean square (RMS) roughness value was determined to be 1.74 nm. Finally, it should be noted that the TEM and AFM results verify that HfO_x_ films without pinholes or cracks were successfully deposited using LNS.

**Figure 3:**
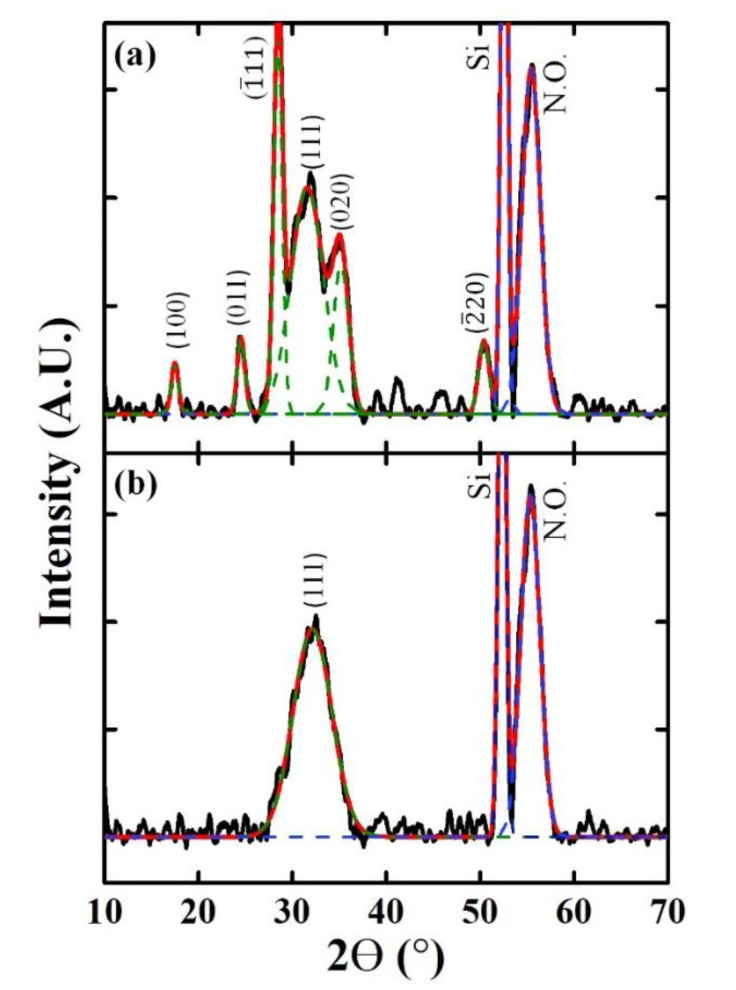
XRD patterns of HfO_x_ films deposited using (**a**) H_2_O and (**b**) LNS. Various peak assignments are shown (N.O. indicates native oxide). The raw data and peak fitting results are shown in black and red, respectively. Green lines indicate peaks assigned to different HfO_x_ film orientations based on deconvolution analysis. Blue lines show Si substrates and interfacial layers. Deconvolution of the XRD patterns was performed using Gaussian functions for the shapes of the resolved peaks.

The crystalline phase of the deposited HfO_x_ films on the Si substrate with optimized process parameters (TEMAH pulse time of 1 s, oxidant pulse time of 0.2 s, and temperature of 300 °C) was identified via GI-XRD. Most of the diffraction peaks of the HfO_x_ films prepared using LNS and H_2_O can be assigned to the monoclinic phase (JCPDS 06-0318), in agreement with the ALD results reported in the literature (Figure 3) [13]. The result was different from that in the case of ZrO_2_, the phase structure of which changed from a tetragonal phase to a monoclinic phase when LNS was used [10]. For the HfO_x_ films prepared with H_2_O, the normal direction of the ($\bar{1}11$) plane at 28.9° was the preferred orientation, as can be clearly seen in the XRD pattern in Figure 3. In addition, the peak assigned to the (111) planes at 31.6° was broad. When LNS was used as the oxidant, a broad peak at around 32.1° appeared, which was similar to the (111) plane observed for the film prepared using H_2_O.

The interplanar distance and crystallite size were calculated from the angular positions of the respective preferred orientations in the XRD patterns; these values are listed in Table 1. The crystallite size of the HfO_x_ films was determined using the Scherrer equation, D = kλ/Bcosθ, where λ is the XRD wavelength (1.5418 Å), k is the shape factor, B is the full width at half maximum of the measured XRD peak in radians, and θ is the Bragg angle. A relatively small crystallite size of 1.7 nm was obtained for the HfO_x_ films deposited using LNS.

**Figure 4:**
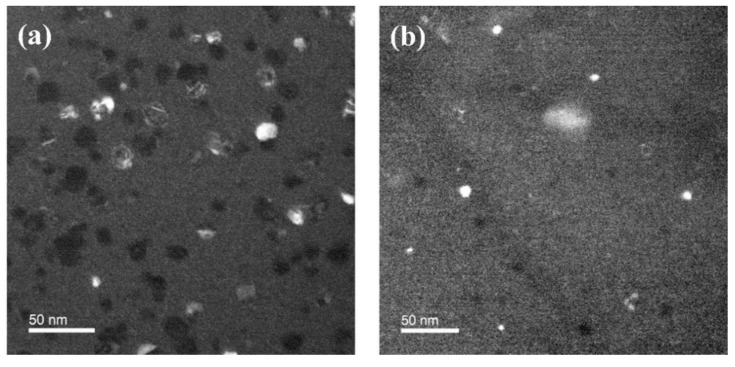
Dark-field TEM plan views of HfO_x_ prepared with (**a**) H_2_O and (**b**) LNS films showing crystallites (black or white areas).

For more details on the difference in the crystallite size, the size of the crystallite was directly observed via dark-field TEM plan views (Figure 4). For the HfO_2_ films fabricated using H_2_O, as shown in Figure 4a, large crystallites were observed, with diameters of more than 20 nm. In the case of the films prepared using LNS (Figure 4b), small crystallites were found with diameters of less than 10 nm. The difference between the XRD and TEM crystallite size results is likely to be related to fact that the shape factor was applied collectively. Nonetheless, the trend of smaller crystallite sizes for the films made using LNS is consistent for the results obtained via the two characterization methods.

### 3.3. Chemical Bonding and Elemental Content of HfO_2_ Films Deposited Using LNS

XPS analyses were carried out to determine the differences between the HfO_x_ films deposited using H_2_O and LNS in terms of their chemical bonding characteristics and compositions. All the analyzed HfO_x_ films were deposited at thicknesses of up to 20 nm on Si substrates. The measured XPS results were deconvoluted using a Shirley background and Gaussian line shapes.

**Figure 5:**
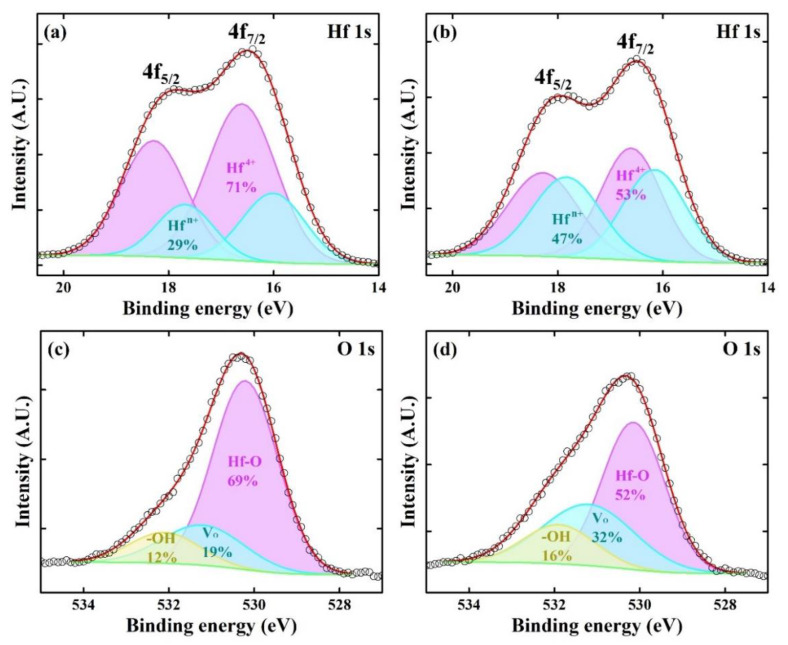
XPS results. Hf 4f spectra of HfO_x_ films fabricated using (**a**) H_2_O and (**b**) LNS. O 1 s spectra of HfO_x_ films fabricated using (**c**) H_2_O and (**d**) LNS. The percentages are estimated values based on deconvolution analysis.

Figure 5a,b show Hf 4f XPS spectra of the HfO_x_ films deposited using H_2_O and LNS, respectively. All the spectra consist of two peaks assigned to 4f_5/2_ and 4f_7/2_ electrons and could be fitted with two sets of the doublet peaks, for which the spin-orbit splitting was 1.68 eV. For the specimens prepared using the H_2_O oxidant (Figure 5a), peaks at 16.60 eV and 18.28 eV forming one of the doublets were assigned to Hf^4+^ 4f_5/2_ and Hf^4+^ 4f_7/2_ of stoichiometric HfO_2_, respectively [14,15]. The doublet at lower energy, 16.00 and 17.68 eV, was assigned to Hf suboxide (HfO_2−x_, 0 < x < 2), that is, the individual peaks in the doublet were assigned to Hf^n+^ 4f_5/2_ and Hf^n+^ 4f_7/2_ (n < 4), respectively [16,17]. It is apparent from the deconvolution results that the fully oxidized Hf^4+^ doublet is much more intense than the suboxidized Hf^n+^ doublet (Figure 5a). As shown in Figure 5b, for the film prepared using LNS, the doublet assigned to stoichiometric HfO_2_ was located at the same binding energy as that of the film prepared using H_2_O (Hf^4+^ 4f_5/2_, 16.60 eV; Hf^4+^ 4f_7/2_, 18.28 eV). Moreover, the positions of the peaks corresponding to Hf^n+^ 4f_5/2_ (16.15 eV) and Hf^n+^ 4f_7/2_ (17.83 eV) were similar to those for the film deposited with H_2_O as the oxidant. However, the Hf^4+^:Hf^n+^ ratio was different for the film prepared using LNS. The ratio of suboxidized Hf^n+^ was significantly increased.

Figure 5c,d show O 1 s spectra of HfO_x_ films deposited using H_2_O and LNS. These were deconvoluted into three components, respectively related to Hf–O bonding in stoichiometric HfO_2_, oxygen vacancies (V_o_), and hydroxyl groups (–OH). In the spectra of both films, three peaks located at 530.1 ± 0.1, 531.2 ± 0.1, and 532.0 ± 0.1 eV, were identified by deconvolutions and assigned to H–O bonding, oxygen vacancies, and hydroxyl groups, respectively [18]. As shown in Figure 5c, for the HfO_x_ film deposited using H_2_O as an oxidant, Hf–O bonding was found to be dominant. However, when the HfO_x_ film was deposited using LNS, the oxygen vacancy and hydroxyl group contents were significantly higher, as shown in Figure 5d. Non-lattice oxygen peaks, such as oxygen vacancy peaks and hydroxyl peaks, contributed to suboxide content in the oxide layer [11,19]. Thus, the O 1 s XPS analysis results are in accord with the Hf 4f XPS analysis results, confirming that the HfO_x_ film deposited using LNS contained more suboxides.

When an HfO_x_ film prepared using LNS was utilized as a resistive switching layer, a higher current density, compared to the HfO_x_ film prepared using H_2_O, was measured in the highly resistive state, indicating that these films formed more current paths [11]. The high current was caused by the increased number of grain boundaries, because of the smaller sizes of the crystallites in the films deposited with the use of LNS. The increase in the non-lattice oxygen (V_o_, –OH) content supplies defect states to the bandgap of oxide films. In addition, as grain boundaries are considered to be reservoirs of oxygen vacancies, the increase in the number of oxygen vacancies is expected with the increase in grain boundaries. Accordingly, the XPS results in Figure 5 are consistent with the previously reported resistive switching results [11], the XRD results as presented in Figure 3, and the TEM results presented Figure 4.

**Figure 6:**
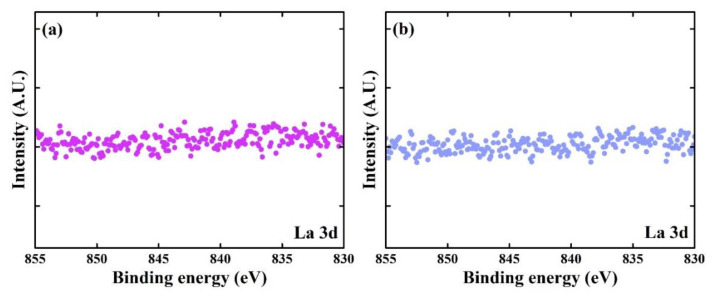
La 3d spectra of HfO_x_ films deposited using (**a**) H_2_O and (**b**) LNS.

The widely reported crystalline phase of HfO_x_ films is monoclinic. However, the doping of metal element into HfO_x_ makes the film orthorhombic phase, showing ferroelectric characteristic. Therefore, to verify the presence or absence of La in the composition of the films, addition XPS data was analyzed. In general, in the XPS analysis for La, spin–orbit peaks of La 3d_5/2_ and La 3d_3/2_ appeared near 835 eV and 850 eV, respectively, and each spin–orbit component was further split via multiplet splitting [20]. However, the XPS data in Figure 6 only show background signals. It indicated that the La content was lower than the detection limit of our XPS analysis method. Thus, the results indicated that the La in the LNS oxidant hardly had very little influence on the chemical composition of the deposited HfO_x_ films. The XRD results indicate that the HfO_x_ existed in the monoclinic phase in the films, and the XPS results show that the La content was below the detection limit. Thus, we conclude that La in LNS is not affected by the chemical reaction with TEMAH, as observed for a ZrO_2_ film prepared using LNS [10]. As a consequence, it can also be concluded that the HfO_x_ deposited using LNS is probably not ferroelectric.

### 3.4. Relationship between Surface Energy and Crystalline Properties

It is plausible that the difference between the surface energies of the film specimens prepared using LNS and H_2_O influences the differences in growth rate and crystallite size. Various research groups have investigated the relationship between surface energy and molecule adsorption. Michiardi et al. reported that when a NiTi alloy underwent oxidation, the total free energy of the alloy increased, and the increase in surface energy caused an increase in the protein adsorption [21]. Moreover, Hayami and Otani reported that in the vapor–liquid–solid process of nanowire growth, for a surface with a higher surface energy, the droplet binding energy was higher [22]. Thus, for droplet binding, the (001) plane, with the highest surface energy, is preferential. In addition, many researchers have reported the correlation of three characteristics of surface energy, orientation, and growth rate. Penn et al. reported that titanium oxide nanoparticles grow rapidly along the [001] direction, driven by the relatively high surface energy of the (001) plane [23].

**Figure 7:**
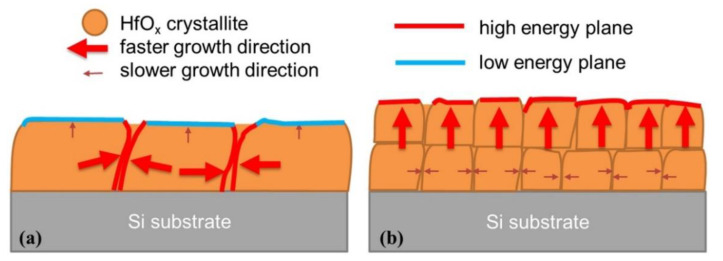
Schematic of HfO_x_ film growth using (**a**) H_2_O and (**b**) LNS.

Finally, we present an explanation for the correlation between the surface energy, growth rate, and crystalline characteristics of the HfO_x_ films. The proposed possible growth mechanisms for the HfO_x_ films are shown in Figure 7.

It is possible that adsorption is more favorable when the surface energy is higher. In HfO_x_ films, the surface energy of the (111) plane is 21% higher than that of the ($\bar{1}11$) plane [24]. When H_2_O is used as an oxidant, HfO_x_ exists mostly as the ($\bar{1}11$) plane, which has the lowest surface energy. The low surface energy prevents the precursor from being absorbed on the surface, and hence vertical growth of the HfO_x_ film was slow. Meanwhile, to reduce the total free energy of the film, the size of the crystallites increases inside the film, eliminating the grain boundaries of with relatively high energies due to lattice mismatch. However, in the case of LNS, a higher surface energy favors the adsorption of the precursor to lower the total energy of the film. It can be hypothesized that differences in precursor adsorption affect the vertical growth rate of HfO_x_ films. In addition, high surface energies affect the crystallization of deposited films. Owing to the high surface energy, many additional nucleation sites existed on the surface of the film. The growth of crystallites at the nucleation sites occurred via the addition of atoms from the precursor. The nuclei grow into crystallites, and the crystallites also offer a surface with high surface energy. Therefore, crystallite growth and the generation of a high-energy surface occurs cyclically. For this reason, a faster HfO_x_ film deposition rate and smaller crystallites were observed when LNS was used in the ALD process.

## 4. Conclusions

Nanocrystalline HfO_x_ films were successfully synthesized by ALD using LNS. The ALD process and film characteristics were compared with those obtained when a conventional oxidant, H_2_O, was used. Typical ALD characteristics such as a self-limiting process, temperature window, and linear dependence of film growth on ALD cycle number were apparent. Interestingly, the growth rate when LNS was used was twice as high as that when H_2_O was used. The XRD results demonstrated that the HfO_x_ films deposited with either LNS or H_2_O both consisted of the monoclinic phase, but there was a difference in the orientation preference. Using LNS, the preferred orientation was (111), which has a higher surface energy than the ($\bar{1}11$) orientation, the preferred orientation of HfO_x_ prepared using H_2_O. The TEM results revealed that the crystallite size of the HfO_x_ film grown using LNS was smaller than that of the film grown using H_2_O. The XPS results showed that the HfO_x_ films prepared using LNS had more suboxides or defects. This was consistent with the fact that the TEM results revealed a higher number of grain boundaries because of the smaller size of the crystallites. Moreover, since it was established via XPS that La dopant atoms were not present in HfO_x_, it is not likely that this material is ferroelectric. Finally, we suggested a growth mechanism model based on the XRD, TEM, and XPS results. It was found that the high surface energy of HfO_x_ films grown using LNS accelerates the adsorption of the precursor and offers more nucleation sites, resulting in small crystallites and a fast growth rate.

## Figures and Tables

**Table 1:** Preferred orientations and crystallite sizes of HfO_x_ films deposited with H_2_O and LNS.

Oxidant	Preferred Orientation	Diffraction Angle of Preferred Orientation	Crystallite Size from XRD (nm)
H_2_O	$(\bar{1}11)$	28.9	7.9
LNS	(111)	32.1	1.7
